# Developmental Changes in Hemodynamic Responses and Cardiovagal Modulation during Isometric Handgrip Exercise

**DOI:** 10.1155/2010/153780

**Published:** 2010-08-29

**Authors:** Styliani Goulopoulou, Bo Fernhall, Jill A. Kanaley

**Affiliations:** ^1^Department of Exercise Science, Syracuse University, Syracuse, NY 13244, USA; ^2^Department of Physiology, Medical College of Georgia, 1120 15th Street, CA-3149, Augusta, GA 30912-3000, USA; ^3^Department of Kinesiology and Community Health, University of Illinois at Urbana Champaign, Champaign, IL 61801, USA; ^4^Department of Nutrition and Exercise Physiology, University of Missouri, Columbia, MO 65211, USA

## Abstract

The purpose of this study was to examine differences in pressor response and cardiovagal modulation during isometric handgrip exercise (IHG) between children and adults. Beat-to-beat heart rate (HR) and blood pressure were measured in 23 prepubertal children and 23 adults at baseline and during IHG. Cardiovagal modulation was quantified by analysis of HR variability. Mean arterial pressure responses to IHG were greater in adults compared to children (*P* < .05) whereas there were no group differences in HR responses (*P* > .05). Children had a greater reduction in cardiovagal modulation in response to IHG compared to adults (*P* < .05). Changes in mean arterial pressure during IHG were correlated with baseline cardiovagal modulation and force produced during isometric contraction (*P* < .05). In conclusion, differences in pressor reflex response between children and adults cannot be solely explained by differences in autonomic modulation and appear to be associated with factors contributing to the force produced during isometric contraction.

## 1. Introduction

Heart rate (HR) variability is a reflection of the interaction between the parasympathetic and sympathetic nervous system influences on the cardiac sinus node [1]. Cross-sectional studies in infants, children, and young adults showed that total HR variability increases up to 6 years of age and declines thereafter, suggesting that autonomic modulation of the sinus node changes with growth and maturation [2–4]. Previous studies have also shown that children have lower resting cardiovagal baroreflex sensitivity (BRS) compared to adolescents and young adults, suggesting that cardiovascular reflex autonomic regulation evolves from childhood to adulthood [3].

The majority of studies investigating the effects of growth on autonomic regulatory mechanisms have focused on measurements obtained during resting conditions [2–4]. The autonomic nervous system, however, plays a paramount role in cardiovascular adaptations to short-term stressors [5]. Therefore, examining the autonomic adjustments to physical stressors may have a greater physiological relevance and provide more useful information than assessing baseline autonomic function. Accordingly, we have previously shown that children 12 years of age had faster HR recovery from exercise associated with greater postexercise HR variability when compared to adolescents aged 15 years [6], suggesting that children had greater parasympathetic reactivation during recovery from exercise compared to adolescents. Others have shown that during standing the percent increase in the low frequency power of HR and systolic blood pressure (BP) variability (i.e., indicators of sympathetic modulation) was higher in adolescents compared to preadolescents [7]. Collectively, these findings suggest that the effects of maturation on autonomic modulation extend to conditions that are characterized by physical stress, such as exercise and orthostatic stimuli.

Isometric exercise provides a convenient and easy way to activate the cardiovascular system and define the role of the autonomic nervous system in the exercise response [8]. Isometric muscle contraction evokes large increases in mean arterial pressure (MAP) and HR with a minor rise in central hemodynamics [9]. These cardiorespiratory responses are mediated by autonomic neural adjustments [10]. Previous studies showed differences in pressor response to isometric handgrip exercise (IHG) between children and adults, with children exhibiting a lower pressor response (smaller BP changes from baseline) compared to adults [11]. To what extent these findings reflect age-associated differences in autonomic regulatory mechanisms in response to isometric contraction is unknown.

Thus, the main purposes of this study were to (1) examine age-associated differences in cardiovascular responses to IHG, and (2) examine differences between children and adults in cardiovagal autonomic modulation and baroreflex sensitivity during isometric contraction. It was hypothesized that children would have a smaller pressor response compared to adults and a greater reduction in cardiovagal modulation during isometric exercise. In the present study, cardiovagal modulation was quantified by analysis of HR variability in the frequency (high frequency power, HF) and time domain (square root of the mean of the sum of the squares of differences between adjacent RR-intervals, RMSSD, and coefficient of variation (CVS)).

## 2. Methods

### 2.1. Subjects

Twenty three healthy children (7–9 years) and 23 healthy young adults (20–25 years) participated in this study. All participants were physically active but did not participate in any organized endurance exercise training program. Exclusion criteria included any medication that alters BP, HR, and autonomic regulation, any known metabolic and cardiovascular diseases, smoking, and hormonal contraceptives. Maturity status was assessed using evaluation of secondary sexual characteristics (Tanner stages for breast and pubic hair) to ensure there were no signs of pubertal development in any of the children. According to this evaluation, all children were prepubertal. All adult females were tested within the first 10 days of their menstrual cycle. The study was approved by the Institutional Review Boards of Syracuse University and SUNY Upstate Medical University and all adult participants, and the parents of the children provided written informed consent and all children provided written assent before testing.

### 2.2. Experimental Design

Two visits were required for the completion of the study. On the first visit, all participants completed a medical history and a physical activity questionnaire and were familiarized with the testing procedures. In addition, measurements of height and weight, and maturity assessment were obtained. On the second visit, the participants completed a questionnaire to verify that they had followed the pretesting instructions. Participants were instructed to refrain from food and caffeinated products for 4 hours and from vigorous exercise and alcohol for 24 hours before the tests. Following a 10 minutes quiet rest, beat-to-beat arterial pressure and continuous electrocardiogram (ECG) were recorded before and during a 3-min IHG task.

### 2.3. Isometric Handgrip Exercise

All participants were tested in the seated position with their elbow flexed at 90°. Maximal isometric force of the dominant hand was measured three times 1-2 minutes apart using a calibrated handgrip dynamometer (Biopac Systems Inc., Santa Barbara, CA). If the trials were not within 10% of each other, additional trials were performed until a plateau was reached. The highest of the 3 values (kg) was defined as the subject's maximal voluntary contraction (MVC). Isometric force was recorded at a frequency of 1000 Hz using a 16-bit data acquisition card (MP100, Biopac Systems, Inc., Santa Barbara, CA). After determination of MVC, all subjects performed a sustained isometric contraction at 30% MVC for 3 minutes. Visual feedback via a computer monitor located 1 m directly in front of the subject and verbal encouragement to maintain the desired force were provided throughout the test. Subjects were instructed to avoid Valsalva maneuver and relax the muscles that did not participate in the isometric contraction.

### 2.4. Beat-to-Beat Hemodynamics

Following 10 minutes of quiet rest (after determination of MVC), continous ECG, and beat-to-beat arterial pressure were recorded for 2 minutes at rest (baseline) and during 3 minutes of IHG. The subjects were tested in the seated position. Heart rate was recorded using a modified CM5 ECG lead, interfaced with data collection and interpretation software (Biopac Systems Inc., CA) at a sampling rate of 1000 Hz. Arterial pressure was measured continuously using a BP finger cuff placed in the middle-finger of the nonexercising hand (Finometer, FMS, the Netherlands). The arterial pressure recordings were performed at a sampling rate of 200 Hz. The Finometer continuously monitors finger arterial pressure using the volume-clamp method of Penaz and performs reconstruction of brachial artery pressure waveform and level from the finger pressure using generalized waveform inverse modeling. This method has been shown to accurately track hemodynamic changes from baseline [12]. Previous studies in both adult and pediatric subjects found that continuous arterial pressure measurements via finger plethysmography slightly underestimated systolic BP (SBP) by 1.9 mmHg and diastolic BP (DBP) by 5.1 mmHg when compared with standard auscultatory techniques [13]. Small within-subject variability (3.8 mmHg for SBP and 4.1 mmHg for DBP) and similar accuracy between children and adults have been also reported, regarding the finger plethysmographic techniques [13]. It was suggested that finger plethysmography is useful for noninvasive assessment of autonomic control and cardiovascular reflexes involving short-term and rapid BP changes in both children and adults [13]. 

### 2.5. Data Analyses

#### 2.5.1. Hemodynamic Variables

Systolic BP, DBP, and MAP were derived from the arterial pressure waveform obtained by the finger plethysmography. Heart rate was calculated from the ECG tracings and stroke volume was calculated from the arterial pressure signal using the arterial pulse wave contour method [14]. Cardiac output was calculated as the product of HR and stroke volume. To account for differences in body size between children and adults, stroke volume and cardiac output are expressed relatively to subjects' body surface area (BSA) and are presented as stroke index and cardiac index, respectively. Total peripheral resistance (TPR) was calculated using the equation: TPR = (MAP/cardiac index) ∗ 80. Baseline values were derived from the average of 3 minutes of data for each hemodynamic variable and IHG values represent the average of the last minute of exercise, during which the maximum values for all subjects were observed.

#### 2.5.2. Heart Rate Variability and Baroreflex Sensitivity Analyses

**Frequency Domain Analysis**

R-R interval time event series were generated from successive HR peaks (WinCPRS, Absolute Aliens Oy, Turku, Finland). Epochs of 120 seconds were analyzed for each condition (i.e., 120 seconds of baseline and the last 120 seconds of IHG measurements) and for each subject. The nonequidistant waveforms were resampled at 5 Hz and passed through a low-pass filter with a cutoff frequency of 0.5 Hz. The spectrum of each signal was calculated using a maximum entropy method (autoregressive modeling). The model order was chosen as the one that minimized Akaike's final prediction error figure of merit. Autoregressive modeling requires a small sample space and therefore, it can be used for analysis of very short-time series [15]. However, this method is vulnerable to nonstationarity (i.e., the degree of signal deviation from baseline) [15]. In this study, stationarity was determined to justify the use of autoregressive modeling (WinCPRS, Absolute Aliens Oy, Turku, Finland). A stationarity value close to zero reflects a stationary signal, while an increase in this value represents an increase in nonstationarity [16]. In this study, mean stationarity of the collected signal at baseline was 0.50 ± 0.02, while mean stationarity for IHG was 0.63 ± 0.03.

Spectral power was expressed as the integrated areas in low (LF: 0.05–0.15 Hz), high (HF: 0.15–0.4 Hz), and total (TP: 0.05–0.4 Hz) frequency ranges. The HF power was used as an index of cardiovagal modulation and was expressed in msec^2^ [17]. In addition, considering the variation in R-R intervals attributable to HF power and the changes in this variation from baseline to IHG, we calculated the coefficient of component variance using the formula: CCV_HF_ = *√*HF power/(mean R-R interval) ∗ 100 (%) [18].

The reproducibility of HR variability measurements has been previously evaluated in our lab in adolescents and young adults and intraclass correlation coefficients were 0.964 and 0.933 (*P* < .05) for LF (msec^2^) and HF (msec^2^), respectively [6].

**Time Domain Analysis**

Heart rate variability was also evaluated in the time domain calculating the mean normal-to-normal R-R intervals and the normal-to-normal R-R intervals differences [15]. These variables were then assessed by statistical analyses providing the following statistics: the standard deviation of the normal-to-normal R-R intervals (SDNN) and the square root of the mean of the sum of the squares of differences between adjacent R-R intervals (RMSSD). In order to control for differences in HR periods from baseline to IHG and between groups, the coefficient of variation (CVS = SDNN/R-R interval) was calculated. All of these measures recognize short-term variations in HR and therefore, estimate HF cyclic components, which are associated with cardiovagal modulation [15].

**Cardiovagal Baroreflex Sensitivity Analysis**

Cardiovagal baroreflex sensitivity (BRS) was determined from the coupling between interbeat intervals and SBP, which were collected via finger plethysmography [19]. This technique has been previously described in [6]. Briefly, beat-to-beat time series of arterial pressure and pulse intervals were searched for sequences of three or more consecutive heart beats in which the pulse intervals and the corresponding SBP increased (WinCPRS, Absolute Aliens Oy, Turku, Finland). These sequences were defined as “baroreflex sequences” and a linear regression line was applied to each of them. The mean slope of all regression lines determined for each subject and for each condition (baseline, IHG) was calculated and taken as a measure of integrated spontaneous BRS. Only sequences with correlations equal or greater than 0.85 were accepted. This measure was used to evaluate the arterial baroreflex modulation of sinus node and its changes from baseline to IHG.

### 2.6. Statistical Analysis

Independent *t*-test was used to examine group differences in subject characteristics (age, height, weight, BMI, BSA, MVC). The HR variability frequency domain and BRS measures were not normally distributed and therefore, they were transformed to natural logarithms (ln ) before statistical analysis. A repeated-measures analysis of covariance (ANCOVA), with one between (group: children versus young adults) and one within (task: baseline versus IHG) factor was carried out to examine differences between children and adults in hemodynamic responses to IHG. Baseline values and BMI were used as covariates to control for the influence of these factors on group responses. In addition, a 2 × 2 ANCOVA with repeated measures was used to determine differences between children and adults in HR variability and BRS responses to IHG. Body surface area and baseline values of HRV were used as covariates to control for their influences on the subjects' responses.

The main assumptions of the analysis of covariance were tested and were met. Specifically, BMI was significantly correlated with all hemodynamic variables, BSA was significantly correlated with all HR variability parameters, and these correlations were the same within each of the populations involved in the study (equal regression slopes). 

Pearson-moment correlations were used to assess the relationship between pressor reflex response to IHG (ΔMAP = MAP_IHG_− MAP_baseline_) and age, BMI, BSA, 30% MVC (absolute intensity in kg), CCV_HF_, and CVS. A stepwise linear regression model of ΔMAP on the above independent variables was performed. Gender was added as a predictor in this model. The significance level of all statistical analyses was set at alpha = 0.05. Data are presented as means ± standard error (SE). Unadjusted and adjusted means as well as unadjusted and adjusted SE are reported for the ANCOVA results.

## 3. Results

### 3.1. Subject Characteristics

Table 1 illustrates the subject characteristics for both groups. As expected, children were younger, shorter, and lighter compared to adults and had lower BMI, BSA, and MVC (*P* < .05). In addition, baseline hemodynamic measures were different between groups. Specifically, children had lower BP (SBP, DBP, MAP), stroke index and cardiac index, and greater HR and TPR at baseline (*P* < .05, Table 2).

### 3.2. Hemodynamic Responses to Handgrip Exercise

**Figure 1:**
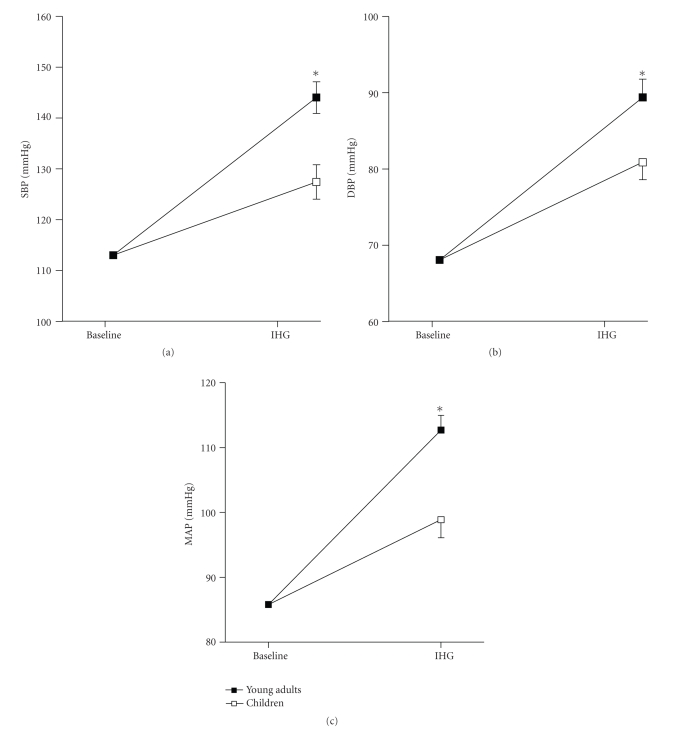
Systolic blood pressure (a), diastolic blood pressure (b) and mean arterial pressure (c) responses to isometric handgrip exercise in children and young adults. SBP: systolic blood pressure; DBP: diastolic blood pressure; MAP: mean arterial pressure; IHG: isometric handgrip exercise. The adjusted means (adjusted to the influence of the covariates) are presented as means ± SE. *P < .05, task by group interaction.

**Figure 2:**
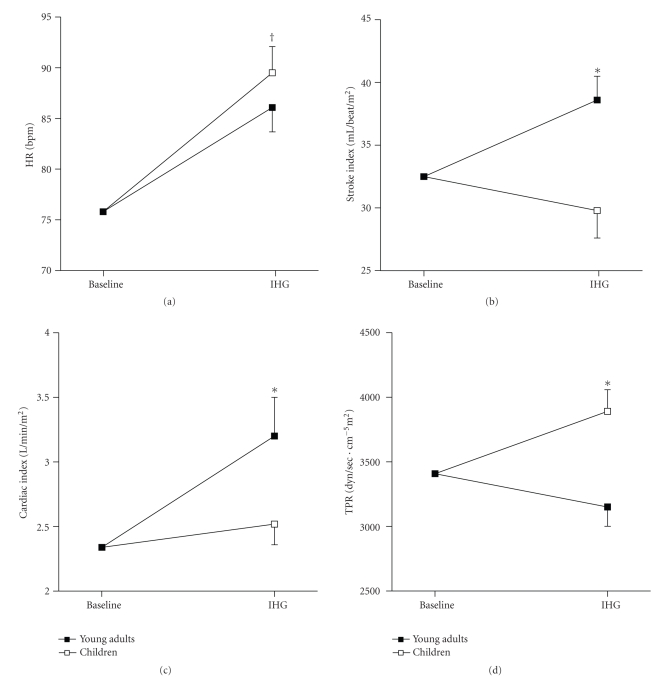
Heart rate (a), stroke index (b), cardiac index (c), and total peripheral resistance (2D) responses to isometric handgrip exercise in children and young adults. HR: heart rate; TPR: total peripheral resistance; IHG: isometric handgrip exercise. The adjusted means (adjusted to the influence of the covariates) are presented as means ± SE. *P < .05, task by group interaction; ^†^ *P* < .05, task effect.

Analysis of covariance was used to determine whether children's hemodynamic responses to IHG differ from those of the adult group, adjusting for baseline measures and BMI. Table 2 displays unadjusted means and SE for all the hemodynamic measurements obtained in the present study. The adjusted means (adjusted to the covariate influence) and SE are presented in Figures 1(a)–1(c) and Figures 2(a)–2(d).

After adjustment for variation in baseline values and BMI, significant group by task interactions were found on SBP, DBP, and MAP, showing that BP significantly increased in response to IHG in both groups but this increase was greater in adults compared to children (*P* < .05, Figures 1(a)–1(c)). Heart rate also increased in response to IHG (*P* < .05) but there were no differences between groups in the magnitude of the response (*P* > .05, Figure 2(a)).

After controlling for baseline values, significant task by group interactions on stroke index, cardiac index, and TPR were also found (*P* < .05, Figures 2(b)–2(d)). Specifically, young adults exhibited an increase in stroke index and cardiac index during IHG (*P* < .05) and no change in TPR (*P* > .05) whereas children showed no change in stroke index (*P* > .05) and an increase in cardiac index and TPR in response to IHG (*P* < .05). Of note, we did not covary for BMI in this analysis because variations in body size were accounted for by expressing cardiac output, stroke volume, and TPR relatively to BSA.

### 3.3. Heart Rate Variability and Baroreflex Sensitivity in Response to Handgrip Exercise

#### 3.3.1. Time Domain Parameters

Children had greater baseline RMSSD and CVS (*P* < .05). An analysis of variance with repeated measures was initially employed to examine differences in HR variability responses to IHG between groups. The results of this analysis showed that R-R intervals decreased in response to IHG in both groups (main effect, *P* < .05), and this decrease was greater in adults compared to children (group by task interaction, *P* < .05). The time domain parameter CVS was reduced in response to IHG only in children (group by task interaction, *P* < .05) whereas RMSSD decreased in both group (main effect, *P* < .05). When we controlled for BSA and baseline values, the task by group interactions presented above were no longer significant (*P* > .05). Table 3 displays unadjusted means and SE, and adjusted means and SE for R-R intervals, RMSSD, and CVS measures at baseline and in response to IHG.

#### 3.3.2. Frequency Domain Analysis

Children had greater baseline ln  HF and CCV_HF_ compared to adults (*P* < .05), but there were no group differences in baseline ln  TP (*P* > .05). Analysis of variance with repeated measures revealed task by group interactions on ln  HF and CCV_HF_. Specifically, children had a significant reduction in ln  HF and CVV_HF_ in response to IHG (*P* < .05), while adults had no change in these variables (*P* > .05). When controlling for BSA and baseline values the task by group interactions presented above were no longer significant (*P* > .05). Table 3 displays unadjusted means and SE, and adjusted means and SE for all frequency domain parameters at baseline and in response to IHG.

#### 3.3.3. Cardiovagal Baroreflex Sensitivity

There were no differences in baseline cardiovagal baroreflex sensitivity between groups (ln  BRS, young adults: 2.6 ± 0.09 msec/mmHg versus children: 2.85 ± 0.10 msec/mmHg, *P* > .05, Table 3). Baroreflex sensitivity was reduced in both groups in response to IHG but the extent of this change was similar between groups (main effect, baseline: 2.74 ± 0.07 msec/mmHg versus IHG: 2.39 ± 0.08 msec/mmHg, *P* < .05).

#### 3.3.4. Correlations

Changes in MAP (ΔMAP) were inversely correlated with baseline CCV_HF_ (*r* = −0.360, *P* < .05) and CVS (*r* = −0.352, *P* < .05) and were positively correlated with BSA (*r* = 0.469, *P* < .01), BMI (*r* = 0.339, *P* < .05), age (*r* = 0.470, *P* < .01), and the force produced at 30% MVC (*r* = 0.526, *P* < .01). After controlling for BSA (partial correlations), the correlations between *Δ*MAP and cardiovagal indices CCV_HF_ and CVS were no longer significant (*P* > .05) whereas the correlation between *Δ*MAP and force produced at 30% MVC remained significant (*P* < .05). The prediction model of the stepwise regression equation indicated that the only significant contributor to variations in ΔMAP was the force produced (30% MVC) during IHG (*R* ^2^ = 0.526, *P* < .001).

## 4. Discussion

The main findings of this study were as follows: (1) children exhibited a lower exercise pressor response compared to young adults, (2) children had a greater reduction in cardiovagal modulation compared to adults during isometric exercise, (3) the differing patterns of autonomic modulation during IHG between children and adults were determined by the greater baseline cardiovagal modulation found in children and their smaller body size, and (4) the magnitude of the pressor response to isometric muscle contraction was directly associated with the force produced at 30% MVC and inversely correlated with baseline cardiovagal modulation.

During isometric exercise, HR, and cardiac output increase, and TPR remains the same or slightly increases, resulting in an increase in SBP, DBP, and MAP [20]. These hemodynamic responses have been well established in adults whereas there is limited data on prepubertal children's responses to isometric contraction. The findings of the present study are in agreement with previous investigations showing differences in pressor response to IHG between children and adults, with children having a lower BP response to isometric exercise [11]. In addition, children showed smaller increases in cardiac index (adults: +26% versus children: +13%) during IHG compared to adults. This lower cardiac index response can be attributed to lower stroke index during exercise, which was only partially compensated for by an elevation in HR levels. In addition, children exhibited greater resting and exercise TPR. In a previous study, Laird and colleagues [21] showed that adolescents increased SBP, DBP, MAP, and cardiac index but had no change in stroke volume and TPR in response to IHG at 25% MVC. These investigators, however, did not collect data on adults and the children participated in their study were older than our prepubertal subjects. Our data are in agreement with previous studies reporting differences in cardiovascular responses to submaximal aerobic exercise between prepubertal children and young adults [22]. In those studies, the differing hemodynamic responses to exercise between children and adults were related to children's smaller hearts and smaller amount of muscle mass being recruited at a given rate of work [22]. The smaller TPR response to exercise found in adults has been previously attributed to greater accumulation of metabolites in adult subjects [23, 24], which contributes to greater vasodilatation [25].

Currently, the mechanisms responsible for the different hemodynamic responses to isometric exercise in prepubertal children are unknown. The cardiovascular responses to isometric contraction are mainly mediated by autonomic adjustments involving sympathetic nervous system activation and vagal withdrawal [10]. Specifically, the early rise in BP is due to tachycardia mediated primarily by a decrease in efferent cardiac vagal activity [9, 26] whereas the rise in BP during sustained contraction is due to an increase in efferent sympathetic activity [9]. Consequently, differing patterns in the pressor reflex during an isometric stimulus may reflect differences in autonomic modulation between children and adults. To the best of our knowledge, this is the first study to examine the role of autonomic modulation in exercise pressor reflex response of pediatric subjects. Although children and adults had similar HR responses to IHG, the reduction in HF power and the time domain parameters of HR variability was greater in children. Pharmacological studies have previously reported that alterations in HR variability reflect changes in parasympathetic modulation of the sinus node [27], which led us to conclude that children had a greater reduction in cardiovagal modulation during isometric contraction compared to adults. Changes in cardiovagal activity (i.e., vagal withdrawal) are critical during isometric contraction because they result in a BP increase (secondary to a tachycardic response) at the onset of the exercise task to match the intensity of the exercise stimulus [9].

After controlling for baseline values, there were no group differences in cardiovagal modulation responses to IHG. This suggests that the greater reduction in cardiovagal activity during exercise in children compared to adults was mainly due to children's elevated cardiovagal activity at baseline, which modulates the extent to which efferent vagal withdrawal will take place under the influence of a physical stress. This finding is in agreement with the law of initial value that states that the outcome of an autonomic response depends on the already existing state of excitation of the autonomic nerves [28]. The present study also found that baseline cardiovagal modulation (CVV_HF_ and CVS) was inversely correlated with changes in MAP from baseline to IHG (*Δ*MAP), indicating that low levels of baseline cardiovagal modulation are related to large exercise pressor reflex responses. This finding confirms the role of vagal modulation in BP responses to isometric contraction in both children and adults. Yet, the relationship between *Δ*MAP and cardiovagal modulation was no longer significant after controlling for body size (i.e., BSA), suggesting that the influences of baseline cardiovagal modulation upon the exercise pressor response are determined by body size.

Although cardiovagal withdrawal plays an important role at the onset of isometric contraction, activation of the sympathetic nervous system is primarily responsible for the rise in BP during sustained isometric activity [9]. In this study, we did not measure sympathetic nervous system activity, and therefore, we cannot state with certainty what the changes in the integrated function of sympathetic modulation were. The BP response to isometric exercise is often used as an index of sympathetic activation during isometric exercise [10]. Accordingly, our results suggest that since children had a lower pressor response to IHG, greater vagal withdrawal, and similar HR responses compared to adults, they probably experienced less cardiac sympathetic modulation during the exercise stimulus. Conversely, since adults had similar HR increases in response to IHG with children, but smaller reductions in cardiovagal modulation, we can speculate that adults had greater cardiac sympathetic modulation. In other words, adults and children attained similar HR responses during isometric contraction through different mechanisms. Specifically, vagal withdrawal might be responsible for increases in HR in children whereas increases in cardiac sympathetic modulation might be the primary mechanism responsible for the tachycardic response during isometric muscle contraction in adults.

Exploring the contribution of reflex neural mechanisms in pressor response to IHG, previous studies reported that differences in metaboreflex sensitivity could explain the age-group differences in pressor response to exercise. Changes in BP during isometric exercise match the intensity of the exercise stimulus, which is directly related to the active skeletal muscle mass as well as to the relative intensity achieved (%MVC) [29]. In the present study, all subjects performed sustained isometric contraction at the same relative intensity (30% MVC). However, the absolute force held at 30% MVC was different between children and adults (children: 5.86 ± 0.35 kg versus young adults: 16.87 ± 1.22 kg), suggesting that adults used greater muscle mass to achieve the same absolute rate of work with children. Greater muscle mass activation would result in greater increases in BP due to greater metabolite accumulation [30]. Indeed, previous studies have shown that adults have greater blood and muscle lactate concentrations during high-intensity exercise compared to children [23, 24]. In a recent study Turley [11] used postexercise ischemia to activate the metaboreceptors and to assess potential differences in metaboreflex function between children and adults. During the 1st minute of postexercise occlusion, children and adults had a similar drop in BP. The greater accumulation of metabolites in adults compared to children [23, 24] and their similar responses immediately following cuff inflation (i.e., ischemia) [11] suggest a more sensitive metaboreflex in the pediatric subjects. In our study, children had a lower BP response to IHG of same relative intensity compared to adults. If indeed children exhibit a more sensitive metaboreflex, their lower pressor response to IHG may be due to age-related differences in other peripheral reflexes mediating the exercise pressor response, such as the mechanoreflex or baroreflex. To the best of our knowledge, currently there is no research on mechanoreflex function in children and therefore, this statement is purely speculative.

In this study, baroreflex sensitivity was reduced in response to IHG and there were no differences in the magnitude of the response between groups. It has been proposed that during exercise arterial baroreflex is reset to a higher operating point by the action of the central command on the central neuron pool receiving baroreceptor afferents [31]. This adaptation allows for a concomitant increase in HR and BP. During isometric exercise central command may also decrease the gain of the integrated baroreflex, depending on the intensity of the exercise stimulus as well as the size of the active muscle mass [29]. The lack of group differences in BRS responses to IHG could explain the absence of group differences in HR responses to this stimulus. Previous studies have shown that children have lower baseline BRS compared to adolescents and young adults but this was not confirmed by our findings [3]. The relative small sample size in our investigation may explain this discrepancy. It is noteworthy that using spontaneous BP and pulse interval sequences to assess baroreflex sensitivity, we only examined the vagal component of the baroreflex whereas the function of the sympathetic arm of the baroreflex during IHG was not investigated. Age-related differences in the sympathetic component of the baroreflex could explain the lower pressor reflex responses suggestive of lower sympathetic activity during exercise in children compared to the adult group.

It should be noted that peripheral autonomic reflexes are not the only determinants of cardiovascular regulation during isometric exercise. Direct action of the central command descending from the higher motor centers on the cardiovascular control areas is also significantly involved in cardiovascular regulation during isometric exercise [9]. This “feed-forward control” mechanism plays a significant role in cardiovascular adaptations at the onset of exercise and is especially important if only a small muscle mass is activated whereas peripheral autonomic reflexes (i.e., metaboreflex and mechanoreflex) become more important in cardiovascular regulation during continued exertion [9]. To the best of our knowledge, currently, there is no research to investigate potential differences in central command during exercise between children and adults and the present study did not assess the contribution of this mechanism to age-group differences. Therefore, we can only hypothesize that age-related differences in central command activation might contribute to differences in cardiovascular adaptations to IHG between children and adults.

Finally, we demonstrated that ΔMAP was related with the force produced at 30% MVC and that this relationship was independent of body size. Furthermore, the force produced at 30% MVC was the only significant predictor of ΔMAP in the regression analysis. This finding suggests that factors that contribute to force production other than body size (i.e., factors other than the size of muscle mass activated) determine the magnitude of the exercise pressor reflex and may be responsible for the differences in exercise pressor reflex seen between children and adults. For example, the type of muscle fibers recruited during isometric contraction by each age group could influence the magnitude of the pressor response. Previous studies suggested that the exercise pressor reflex is elicited selectively from activation of the fast twitch fibers [32]. Children have lower neuromuscular activation (recruitment and firing rate of motor units) during sustained isometric contraction compared to adolescents and adults [33]. Therefore, children may also have less capability to activate Type II motor units than adults during muscular contractions. Indeed, Halin et al. [34] reported that adults recruited a greater number of Type II fibers during isometric contraction compared to children. According to these findings, the greater pressor response seen in adults in the present study may be attributed to the type of muscle fibers recruited by this group.

## 5. Limitations

In this study, cardiovagal autonomic regulation was solely assessed with analysis of HR variability. Spectral analysis of HR variability provides information about parasympathetic modulation but does not give us insight into the status of the tonic stimulus [35]. Therefore, our conclusions are limited to the effects of IHG on vagal influences on the modulations of HR. Consequently, inferences cannot be made regarding the tone of the parasympathetic nervous system during IHG. Moreover, we examined the exercise pressor reflex as a whole, involving all peripheral reflexes, and therefore, we cannot draw any conclusions regarding the contribution of each reflex to pressor response. Further, all measures were collected during spontaneous breathing. Respiratory frequencies and tidal volume may affect the HF power of HR variability [36]. Therefore, in adult studies respiration is usually controlled during HR variability measurements by instructing the subjects to breathe at a specific rate. In children, however, uncontrolled breathing may be a better method because it reduces psychological strain [7]. Studies in our laboratory (unpublished data) found that respiratory rate did not significantly change from resting conditions (baseline) to isometric handgrip exercise in middle-aged adults.

In conclusion, the findings of this study suggest that baseline cardiovagal modulation contributes to the magnitude of the pressor reflex response and determines the extent of cardiovagal autonomic adjustments during forearm isometric contraction. Yet, differences in muscle pressor reflex response between children and adults cannot be solely explained by differences in autonomic modulation between groups and appear to be associated with factors determining the absolute force produced during submaximal isometric contraction. 

## Figures and Tables

**Table 1:** Subject characteristics.

Variables	Young adults	Children
No (males/females)	23 (13/10)	21 (12/9)
Age (yr)	22.0 ± 0.3	8.3 ± 0.2*
Height (cm)	171.4 ± 1.7	132.6 ± 1.8*
Weight (kg)	76.9 ± 3.7	34.3 ± 2.7*
BMI (kg/m^2^)	26 ± 1	19 ± 1*
BSA (m^2^)	1.85 ± 0.04	1.05 ± 0.04*
MVC (kg)	56.2 ± 4.1	19.8 ± 1.3*

Values are means ± SE.

BMI: body mass index; BSA: body surface area; MVC: maximal voluntary contraction.

*P < .05, group differences.

**Table 2:** Hemodynamic variables (unadjusted means) at baseline and in response to handgrip exercise.

	Young adults		Children	
Variables	Baseline	IHG	Baseline	IHG
SBP (mmHg)	117.5 ± 2.2	148.6 ± 3.7	107.9 ± 2.0^‡^	122.1 ± 2.7^∗†^
DBP (mmHg)	71.3 ± 1.2	91.9 ± 2.1	64.6 ± 2.0^‡^	78.1 ± 2.5^∗†^
MAP (mmHg)	88.5 ± 1.5	114.9 ± 2.7	82.8 ± 2.4^‡^	96.4 ± 2.4^∗†^
HR (bpm)	68.8 ± 2.0	82.6 ± 2.1	84.0 ± 2.0^‡^	93.5 ± 2.2*
Cardiac index (L/min/m^2^)	3.1 ± 0.1	3.9 ± 0.2	1.5 ± 0.06^‡^	1.7 ± 0.07^∗†^
Stroke index (mL/beat/m^2^)	45.5 ± 1.5	49.0 ± 1.4	17.6 ± 0.6^‡^	17.9 ± 0.7^∗†^
TPR (dyn/sec·cm^−5^·m^2^)	2344 ± 90	2358 ± 85	4631 ± 224^‡^	4802 ± 223

Means ± SE, values are not adjusted for the influence of the covariates.

*P < .05, measures during IHG are different from baseline (main effect); ^†^ *P* < .05, task by group interaction; ^‡^ *P* < .05, group differences in baseline hemodynamic variables.

SBP: systolic blood pressure; DBP: diastolic blood pressure; MAP: mean arterial pressure; HR: heart rate; TPR: total peripheral resistance.

**Table 3:** Cardiovagal modulation and baroreflex sensitivity in response to isometric handgrip exercise.

Parameters	Baseline	Handgrip Exercise	
		Obtained	Adjusted
RR-interval, msec			
Young adults	879 ± 24	743 ± 18*	689 ± 18
Children	727 ± 26	660 ± 20^∗†^	722 ± 19
ln TP, msec^2^			
Young adults	8.2 ± 0.1	7.6 ± 0.2*	7.4 ± 0.2
Children	8.4 ± 0.1	7.9 ± 0.1*	8.0 ± 0.3
ln HF, msec^2^			
Young adults	6.1 ± 0.2	6.0 ± 0.2*	6.1 ± 0.3
Children	6.9 ± 0.2	6.3 ± 0.2^∗†^	6.3 ± 0.3
CCV_HF_			
Young adults	2.7 ± 0.3	3.0 ± 0.3	3.4 ± 0.6
Children	4.8 ± 0.3	3.8 ± 0.4^†^	3.4 ± 0.7
RMSSD, msec			
Young adults	47.0 ± 5.1	40.4 ± 4.7*	40.6 ± 7.9
Children	64.3 ± 5.9	42.7 ± 5.4*	41.4 ± 8.6
CVS, %			
Young adults	5.2 ± 0.5	5.5 ± 0.7*	5.7 ± 1.2
Children	8.8 ± 0.6	6.4 ± 0.8^∗†^	5.9 ± 1.3
ln BRS, msec/mmHg			
Young adults	2.60 ± 0.09	2.26 ± 0.11	2.38 ± 0.15
Children	2.85 ± 0.10	2.60 ± 0.12	2.44 ± 0.16

Means ± SE. *P < .05, responses to IHG are different from baseline (main effect); ^†^ *P* < .05, task by group interaction.

ln natural logarithm; TP: total power; HF: high frequency; CCV: coefficient of component variance; RMSSD: the square root of the mean of the sum of the squares of differences between adjacent R-R intervals; CVS: coefficient of variation; BRS: baroreflex sensitivity.

## References

[1] Malliani A, Pagani M, Lombardi F, Cerutti S (1991). Cardiovascular neural regulation explored in the frequency domain. *Circulation*.

[2] Finley JP, Nugent ST (1995). Heart rate variability in infants, children and young adults. *Journal of the Autonomic Nervous System*.

[3] Lenard Z, Studinger P, Mersich B, Kocsis L, Kollai M (2004). Maturation of cardiovagal autonomic function from childhood to young adult age. *Circulation*.

[4] Pikkujämsä SM, Mäkikallio TH, Sourander LB (1999). Cardiac interbeat interval dynamics from childhood to senescence: comparison of conventional and new measures based on fractals and chaos theory. *Circulation*.

[5] Rowell LB (1993). *Human Cardiovascular Control*.

[6] Goulopoulou S, Heffernan KS, Fernhall B, Yates G, Baxter-Jones ADG, Unnithan VB (2006). Heart rate variability during recovery from a Wingate test in adolescent males. *Medicine and Science in Sports and Exercise*.

[7] Tanaka H, Borres M, Thulesius O, Tamai H, Ericson MO, Lindblad L-E (2000). Blood pressure and cardiovascular autonomic function in healthy children and adolescents. *Journal of Pediatrics*.

[8] Khurana RK, Setty A (1996). The value of the isometric hand-grip test—studies in various autonomic disorders. *Clinical Autonomic Research*.

[9] Kjaer M, Secher NH (1992). Neural influence on cardiovascular and endocrine responses to static exercise in humans. *Sports Medicine*.

[10] Seals DR, Chase PB, Taylor JA (1988). Autonomic mediation of the pressor responses to isometric exercise in humans. *Journal of Applied Physiology*.

[11] Turley KR (2005). The chemoreflex: adult versus child comparison. *Medicine and Science in Sports and Exercise*.

[12] Parati G, Casadei R, Groppelli A, Di Rienzo M, Mancia G (1989). Comparison of finger and intra-arterial blood pressure monitoring at rest and during laboratory testing. *Hypertension*.

[13] Tanaka H, Thulesius O, Yamaguchi H, Mino M, Konishi K (1994). Continuous non-invasive finger blood pressure monitoring in children. *Acta Paediatrica*.

[14] Brock-Utne JG, Blake GTW, Bosenberg AT, Gaffin SL, Humphrey D, Downing JW (1984). An evaluation of the pulse-contour method of measuring cardiac output. *South African Medical Journal*.

[15] (1996). Heart rate variability: standards of measurement, physiological interpretation, and clinical use. Task Force of the European Society of Cardiology and the North American Society of Pacing and Electrophysiology. *Circulation*.

[16] Heffernan KS, Jae SY, Vieira VJ (2009). C-reactive protein and cardiac vagal activity following resistance exercise training in young African-American and white men. *American Journal of Physiology*.

[17] Byrne EA, Fleg JL, Vaitkevicius PV, Wright J, Porges SW (1996). Role of aerobic capacity and body mass index in the age-associated decline in heart rate variability. *Journal of Applied Physiology*.

[18] Hayano J, Yamada M, Sakakibara Y (1990). Short- and long-term effects of cigarette smoking on heart rate variability. *American Journal of Cardiology*.

[19] Goulopoulou S, Fernhall B, Kanaley JA (2009). Hemodynamic responses and linear and non-linear dynamics of cardiovascular autonomic regulation following supramaximal exercise. *European Journal of Applied Physiology*.

[20] Turley KR (1997). Cardiovascular responses to exercise in children. *Sports Medicine*.

[21] Laird WP, Fixler DE, Huffines FD (1979). Cardiovascular response to isometric exercise in normal adolescents. *Circulation*.

[22] Turley KR, Wilmore JH (1997). Cardiovascular responses to treadmill and cycle ergometer exercise in children and adults. *Journal of Applied Physiology*.

[23] Cooper DM, Barstow TJ (1996). Magnetic resonance imaging and spectroscopy in studying exercise in children. *Exercise and Sport Sciences Reviews*.

[24] Eriksson BO, Karlsson J, Saltin B (1971). Muscle metabolites during exercise in pubertal boys. *Acta Paediatrica Scandinavica, Supplement*.

[25] Nottin S, Vinet A, Stecken F (2002). Central and peripheral cardiovascular adaptations to exercise in endurance-trained children. *Acta Physiologica Scandinavica*.

[26] Flessas AP, Ryan TJ (1983). Atropine-induced cardioacceleration in patients on chronic propranolol therapy: comparison with the positive chronotropic effect of isometric exercise. *American Heart Journal*.

[27] Fagraeus L, Linnarsson D (1976). Autonomic origin of heart rate fluctuations at the onset of muscular exercise. *Journal of Applied Physiology*.

[28] Wilder J (1967). *Stimulus and Response: The Law of Initial Value*.

[29] Iellamo F, Massaro M, Raimondi G, Peruzzi G, Legramante JM (1999). Role of muscular factors in cardiorespiratory responses to static exercise: contribution of reflex mechanisms. *Journal of Applied Physiology*.

[30] Lewis SF, Snell PG, Taylor WF (1985). Role of muscle mass and mode of contraction in circulatory responses to exercise. *Journal of Applied Physiology*.

[31] Iellamo F, Hughson RL, Castrucci F (1994). Evaluation of spontaneous baroreflex modulation of sinus node during isometric exercise in healthy humans. *American Journal of Physiology*.

[32] Fallentin N, Sidenius B, Jorgensen K (1985). Blood pressure, heart rate and EMG in low level static contractions. *Acta Physiologica Scandinavica*.

[33] Paasuke M, Ereline J, Gapeyeva H (2000). Twitch contraction properties of plantar flexor muscles in pre- and post-pubertal boys and men. *European Journal of Applied Physiology*.

[34] Halin R, Germain P, Bercier S, Kapitaniak B, Buttelli O (2003). Neuromuscular response of young boys versus men during sustained maximal contraction. *Medicine and Science in Sports and Exercise*.

[35] Malik M, Camm AJ (1993). Components of heart rate variability—what they really mean and what we really measure. *American Journal of Cardiology*.

[36] Brown TE, Beightol LA, Koh J, Eckberg DL (1993). Important influence of respiration on human R-R interval power spectra is largely ignored. *Journal of Applied Physiology*.

